# Sigma-Class Glutathione Transferases (GSTσ): A New Target with Potential for Helminth Control

**DOI:** 10.3390/tropicalmed9040085

**Published:** 2024-04-16

**Authors:** Lluvia de Carolina Sánchez Pérez, Rafael A. Zubillaga, Ponciano García-Gutiérrez, Abraham Landa

**Affiliations:** 1 Departamento de Química, Universidad Autónoma Metropolitana-Iztapalapa, Mexico City C.P. 09310, Mexico; lluvia_sanper@xanum.uam.mx (L.d.C.S.P.); pgarcia@xanum.uam.mx (P.G.-G.); 2 Departamento de Microbiología y Parasitología, Facultad de Medicina, Universidad Nacional Autónoma de México, Ciudad Universitaria, Mexico City C.P. 04510, Mexico

**Keywords:** GST sigma, prostaglandin, resistance, antigens, vaccination, helminths

## Abstract

Glutathione transferases (GSTs EC 2.5.1.18) are critical components of phase II metabolism, instrumental in xenobiotics’ metabolism. Their primary function involves conjugating glutathione to both endogenous and exogenous toxic compounds, which increases their solubility and enables their ejection from cells. They also play a role in the transport of non-substrate compounds and immunomodulation, aiding in parasite establishment within its host. The cytosolic GST subfamily is the most abundant and diverse in helminths, and sigma-class GST (GSTσ) belongs to it. This review focuses on three key functions of GSTσ: serving as a detoxifying agent that provides drug resistance, functioning as an immune system modulator through its involvement in prostaglandins synthesis, and acting as a vaccine antigen.

## 1. Introduction

Detoxification is an important enzyme function, and organisms have evolved complex and efficient mechanisms to perform it. One such mechanism is xenobiotic metabolism, existing in three phases: bioactivation, conjugation, and transport [1]. Xenobiotics are chemical substances that are not nutrients and are not necessary for maintaining homeostasis or normal physiological and biochemical functions. Some can be toxic, such as drugs, food additives, and heavy metals [2].

Different families of enzymes perform this metabolic process through three phases. In phase I, cytochrome P450 (CPY) enzymes are predominantly used. Phase II involves glutathione transferases (GSTs), a crucial group responsible for conjugating reduced glutathione with toxic compounds to enhance their solubility and ease excretion. Lastly, in phase III, the compounds are expelled from cells through transporters such as ABC [3]. This review focuses on sigma-class GST (GSTσ), discussing studies on and contributions to this class of enzymes in various organisms to better understand its functions. The aim is to show its potential for controlling diseases caused by helminths, with a focus on developing environmentally friendly and safe products for humans.

## 2. Glutathione Transferases (GSTs)

GSTs (EC 2.5.1.18) are a multifunctional superfamily of enzymes found in all aerobic organisms [4]. They primarily catalyze the conjugation of the thiolate ion of reduced glutathione (GSH) to the electrophilic centers of hydrophobic organic molecules [3,5]. This process is essentially an evolutionary adaptation for the efficient elimination of numerous potentially toxic chemicals [6].

GSTs assist in the metabolism of harmful endogenous compounds, such as free radicals and reactive oxygen species. They also process xenobiotics, including aldehydes, quinones, epoxides, hydroperoxides, drugs, carcinogens, and environmental pollutants. Furthermore, GSTs play a role in the sequestration and transport of these compounds [7,8]. Within the GST family in helminths, there are three subfamilies: the cytosolic subfamily, which is abundant in cells; the mitochondrial subfamily; and the microsomal subfamily (MAPEG). The latter is associated with the membrane and is involved in the metabolism of eicosanoids and glutathione. To date, these subfamilies have been identified and described in mammals, reptiles, plants, mollusks, insects, helminths, and bacteria [9,10,11].

GSTs perform several functions, which include conjugation with glutathione (GSH), the biosynthesis of key biological mediators, the modulation of cellular signals, and the regulation of calcium channels. They also transport non-substrate molecules, including bilirubin, the heme group, steroids, hormones, and bile salts [10,12]. Moreover, they play an important role in the catabolism of tyrosine and catalyze the isomerization reactions essential for the synthesis of prostaglandins and steroids. GSTs also aid in the removal of reactive oxygen species and the regeneration of S-thiolated proteins, offering protection against oxidative cell damage [10,13]. Table 1 shows the core functions of the three main GST families, along with the types that each one possesses, based on primary structure similarities, substrate specificity, inhibition properties, and isoelectric point [14]. For a more in-depth understanding, we recommend reviewing [15].

The various classes of GSTs have multiple isoforms that serve the same function, with slight differences in their primary structure and subsequent electrical charge. This has been observed across a range of organisms, including rats, humans, plants, parasites, and insects. The presence of multiple isoforms provides biological and adaptative advantages, allowing organisms to biotransform and eliminate a broader of toxic com-pounds, which would be impossible with just one isoform [14]. The level of sequence identity for enzymes within the same class is typically high, around 50%, with in a species. However, this drops to 20–40% between different classes [24,25]. The most exten-sively studied GST family is the cytosolic family, largely due to its significant role in human pharmacology.

The structural features of most cytosolic GST classes—such as their compact folds, conserved structure, well-defined domains, and binding cavities at G, H, and L sites—make them easier to study [10].

### 2.1. Cytosolic GST (cGST)

Thirteen classes of GSTs in animals and plants are displayed in Table 1. Mammals boast the most classifications, with seven, while plants have six. These have been identified in insects, helminths, mollusks, and fungi [13,26]. The cGST subfamily, which contributes to approximately 3–4% of a cell’s total soluble protein, underlines its significance. This subfamily is further divided based on structural similarity and the type of catalytic residue at the GSH binding site forming the GS^-^ thiolate anion. The alpha, mu, pi, and sigma classes, which seemingly evolved more recently, activate GSH using a tyrosine near the N-terminus, whereas the older theta, omega, and zeta classes utilize serine or cysteine for activation [27]. The quaternary structure of cGST is composed of two monomers, each weighing between 23 and 28 kDa, forming a roughly 50 kDa dimer. The dimer can feature identical (homodimer) or different (heterodimer) subunits from the same class. The dimeric configuration is crucial for enhancing protein stability and ensuring the proper active site structure for efficient catalysis [28,29].

Each monomeric unit of cGST integrates an N-terminal domain harboring a GSH binding site (G site) composed of four mixed β strands, of which the first, second, and fourth point in the same direction, while the third is antiparallel. This domain also includes three α helices and is arranged in a βαβαββα format, resembling that of thioredoxin [3]. The C-terminal domain contains a hydrophobic binding site for electrophilic substrates (H-site) with only α helices, the count of which varies between five and six according to the class. Notably, the H-site displays considerable structural variability among classes, along with differing specificity and substrate recognition attributes, given its plasticity and flexibility. This contributes to the enzyme’s catalytic promiscuity, enabling it to bind to various electrophilic substrates [8]. These structural differences aid cells in eliminating a more diverse range of toxic compounds, as the binding site (H) affinity differs across classes [30].

#### 2.1.1. Glutathione (GSH)

Glutathione, a water-soluble tripeptide composed of glutamic acid, cysteine, and glycine (L-g-glutamyl-L-cysteinyl-glycine), is an essential factor in maintaining cellular REDOX balance and is found throughout the body [31]. This molecule exists in reduced (GSH) and oxidized (GSSG) states, as well as being bound to proteins. The free form predominantly exists within cells, serving as a reductant or substrate in protection against oxidative and xenobiotic stresses [2]. Meticulously evolved over time, glutathione performs many functions, with one of the most significant being the detoxification of foreign compounds (xenobiotics) through enzyme-regulated processes involving GST [31,32]. If these potentially hazardous xenobiotics were not neutralized by GSH, they could react directly with DNA, RNA, lipids, or proteins within cells, causing significant damage. Additionally, the introduction of GSH to reactive electrophiles tags the R group with the tripeptide, setting it up for recognition by ATP-dependent GSH export pumps, which expel such conjugates from the cell [33,34].

#### 2.1.2. Catalytic Mechanism of the Conjugation Reaction

To catalyze the conjugation reaction, certain physicochemical alterations are needed. First, a structural rearrangement must take place, characterized by typical conformational changes induced by the substrate. Second, the ionization of reduced GSH, which produces the thiolate anion (GS^-^), is required. The binding of GSH to GST has been found to decrease the pKa value of the -SH group at the G site [35,36]. This reduction in the pKa of the thiol group improves its ionization capability, thereby generating a more potent nucleophile (RS^-^ versus RSH). This enhanced nucleophile can then interact more effectively with the electrophilic substrate at the H-site at a neutral pH. This accounts for the high conservation of tyrosine and why its -OH group acts as the acceptor in the hydrogen bond formed between the G site and the GSH [4,18].

#### 2.1.3. Substrates and Specificity

A variety of substrates are currently utilized, along with other parameters, to ascertain the specificity of a GST and its classification. Many GSTs demonstrate enzymatic activity toward 1-chloro-2,4-dinitrobenzene (CDNB), which serves as a standard substrate. However, various other substrates have been employed, including 1,2-dichloro-4-nitrobenzene (DCNB), ethacrynic acid, lipid hydroperoxides, and reactive carbonyls. Furthermore, inhibitors have been used as class markers, such as bromosulfophthalein for the alpha class, cibacron blue, and triphenyltin chloride for the mu class. Other substances employed are hematin-related compounds and bile acids, which help to define their class and specificity (Table 2) [37,38].

Although various substrates have been used to identify new GSTs, the specificity or inhibition degree of each enzyme for the same compound can differ, even if they belong to the same class. A comparative study of activities with 4-hydroxyalkenal (HNEs) and other activated alkenes has provided an understanding of certain GSTs’ active site properties. It has been proven that the specificity of 15 GSTs against 4-hydroxyalkenals (HNEs) is not related to their class membership; the higher the substrate’s hydrophobicity, the greater the binding free energy. The free-energy differences are estimated from their specificity constants (*k_cat_*/*K_M_*) [35]. For some enzymes, the active site’s steric limitations seem to offset the increased binding energy attributed to the increase in substrate chain length.

It is worth noting how glutathione (GSH) interacts with cytosolic GSTs. The tripeptide adopts an extended configuration upon binding to the enzyme at a β-strand end, with the cysteine sulfur pointing toward its monomer’s core, where it interacts with the hydrogen-bond donor. For example, for recombinant GSTσ from *Taenia solium* (TsMσGST), the formation of a hydrogen bond between the GS^-^ sulfur and the Tyr14 hydroxyl has been observed [44]. In the case of theta- and zeta-class GSTs, the hydroxyl-group donor originates from a serine [19].

The most conserved region across all GSTs begins with a cis-proline residue before the β3 strand and continues through the α3 helix. This region plays a crucial role in binding the γ-glutamyl residue of GSH by providing hydrogen-bond interactions with glutamine or glutamate, followed by a serine or threonine located at the turn between the β4 strand and the α3 helix [5,55].

## 3. Structural and Functional Generalities of GSTσ

GSTσ was initially described in various species, including squid, humans, mice, rats, birds, insects, helminths, mollusks, and frogs [9]. According to Ji and colleagues [56], GSTσ evolutionarily diverged first from the same ancestral precursor as the alpha, mu, and pi classes. Its three-dimensional structure parallels that of other cytosolic GSTs, although a much shorter C-terminal domain gives it a more open active site. The N-terminal domain consists of α-helices and β-strands, which are smaller than those in the C-terminal domain. The latter, composed solely of α helices, contains most of the residues forming the H-site cavity [57].

The specific architecture of each GST’s catalytic site allows for unique functions besides detoxification. For example, the rat hematopoietic prostaglandin D synthase (H-PGDS) enzyme, which is a type of GSTσ, solely catalyzes the isomerization of PGH_2_ to PGD_2_. A similar function is demonstrated by the GSTσ of *Schistosoma mansoni* but not by the GSTσ of squid, which produces a mixture of PGD_2_, PGE_2_, and PGF_2α_ from the same substrate [58,59,60]. In mammals, prostaglandins mediate various functions, including immune-response modulation, sleep promotion, hormone release, and vasodilation. The generation of diverse products by a single GST class could be attributed to an organism’s need to regulate its physiological activities or to the need to primarily evade the host’s immune response.

A noted variation occurs in the FhGST-S1 variant of *Fasciola hepatica*, which exhibits a disulfide bond between the cysteines at positions 26 and 196. This bond is crucial for the formation of the active site and is thought to help stabilize the enzyme when released into the host’s stomach. It is worth mentioning that FhGST-S1 is expressed in the Fasciola’s extracellular vesicles and can be released to modulate the host’s immune response [57].

**Figure 1:**
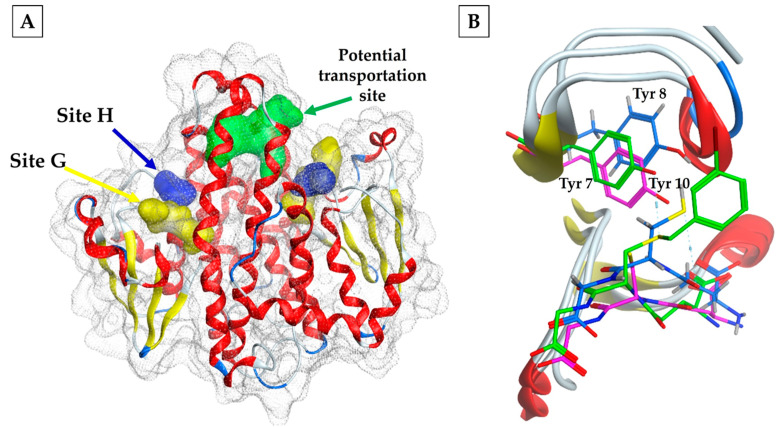
(**A**) Identification of the G (yellow) and H (blue) sites and a third site with the potential to transport non-substrate compounds (green). (**B**) Superposition of three crystallographic structures of GSTσ, where the conservation of Tyr is observed as the residue responsible for activating the thiol group of GSH (2GSQ.pdb (green), GSTσ in squid; 2WB9.pdb (magenta), FhGST-S1; 3VPQ.pdb (blue), and bmGST1.

Conversely, there is a third potential binding site named “L” (Figure 1A). This site is a hydrophobic cavity located approximately 14 Å from the hydroxyl group of Tyr-7 of the active site, that binds non-catalytic ligands, such as synthetic organic compounds, reactive oxylipins, phenols, flavonoids, and unstable intermediates. This binding process results in the sequestration or storage of these compounds or their transport to specified sites. Notably, this binding may inhibit the catalytic activity of the GSTs either competitively or non-competitively [61]. In the case of the squid enzyme, the L site transports glutathione with its conjugates, as revealed by the crystal structure of the protein complexed with S-(3-iodobenzyl) glutathione [56].

Electrostatic interactions near the active site are considered significant. As commonly observed, GSH binds consistently among all cytosolic GSTs, creating a hydrogen bond between the cysteine’s sulfhydryl group and the catalytic tyrosine; this residue is highly conserved in sigma-class GSTs, as well as in the alpha, mu, and pi classes. The preservation of Tyr as a hydrogen-bond donor has been identified in the GSTσ in several organisms, as demonstrated by bmGST1 in the insect *Bombix mori*, FhGST-S1 in *Fasciola hepatica*, and glutathione transferase in the squid *Loligo vulgaris*, where Tyr 8, Tyr 10, and Tyr 7 form the hydrogen bond, respectively [57,62,63] (Figure 1B).

In other cGSTs, a different catalytic residue is present, such as Ser in the theta and zeta classes or Cys in the omega class. Ser plays a role similar to that of Tyr in alpha-class GSTs, such as mu, pi, and sigma. Meanwhile, Cys, in the omega class, forms a mixed disulfide with GSH, resulting in variations in their catalysis mechanism. An interesting characteristic of GSTσ is its low activity against the universal substrate for GST, 1-chloro-2,4-dinitrobenzene (CDNB), but high activity against 4-hydroxynonenal (HNE). The latter is a cytotoxic peroxidation compound produced by oxidative stress [46]. For *F. hepatica*, two isoforms of GSTσ, FhGST-S1 and FhGST-S2, are reported. FhGST-S2 lacks total activity against the most common GST substrates, except for reactive aldehydes such as trans-2-Nonenal and trans-2,4-decadienal. This suggests a unique role for this isoform [42], as shown in Table 2.

### 3.1. Role of GSTσ in Helminths

Helminths have co-evolved with their host species, developing adaptive mechanisms for survival. For instance, they alter the genes that code for their actin isoforms and activate their detoxification enzymatic systems. These systems decrease the effects of drugs and, consequently, increase resistance [64]. Among helminths, GSTs are the predominant enzymes involved in the phase II metabolism of xenobiotics [12]. This prevalence is attributed to the parasites’ low expression of phase I detoxification enzymes, such as the cytochrome P450 family and glutathione peroxidase. Consequently, GST is the lead detoxification system in helminths [65,66].

#### GSTσ as Prostaglandin Synthase in Helminths

Recently, it was discovered that helminth GSTσ can also synthesize prostaglandins, which regulate cellular homeostasis and multiple physiological responses in humans. The main types of prostaglandins are PGD_2_, PGE_2_, PGF_2_, TXA_2_ (thromboxane), and PGI_2_ (prostacyclin). The first two are produced by GSTσ in helminths and have distinct functions. PGD_2_ triggers sleepiness during parasitic infection, restrains the migration of the host’s epidermal Langerhans cells (LC), and instigates programmed cell death. Conversely, PGE_2_ acts as a powerful immunomodulator, causing inflammation, fever, headaches, and bodily aches, reducing cytokine production, enabling signal coupling during phagocytosis, and enhancing the parasite’s ability to penetrate host cells [9,67]. It is recognized that prostaglandins are crucial for the initiation and progress of parasitosis.

In the context of helminth infections, their prostaglandins impact their growth and reproduction, as well as manipulate the host’s immune system to their advantage [39,68]. Notably, the prostaglandins PGD_2_ and PGE_2_ are found throughout various stages of the parasite’s development, synthesized by the helminth’s GSTσ and the microsomal GST (MAPEG), respectively. High concentrations of PGD_2_ have been found in non-embryonic eggs, indicating a potential role in egg development [40]. Prostaglandin production similarly modulates the host’s immune response in the sexually immature metacercariae stage.

The presence of GSTσ in tissues that interact directly with the host suggests that prostaglandin synthesis is vital for the parasite’s survival and reproduction, contributing to changes in the parasite’s pathophysiology and its ability to invade and establish itself. Specifically, PGE_2_ also impacts the fertilization and survival of eggs. Researchers have discovered that GSTσ from *Schistosoma mansoni* (Sm28GST) produces PGD_2_ upon penetrating the host’s skin. PGD_2_ inhibits the migration of LCs to the skin’s surface. Given LCs’ essential role in establishing skin immunity by triggering immune responses in the presence of antigens, this shows how *S. mansoni* has developed a strategy to neutralize the host’s immune response [69]. Moreover, PGD_2_ obstructs the LCs’ departure from the epidermis and the accumulation of dendritic cells in the lymph nodes, responsible for increasing the immune response [70]. This suppressive effect is mediated by soluble lipophilic factors released by the parasites, not host-derived anti-inflammatory cytokines, emphasizing PGD_2_’s role in stalling LC migration. These findings highlight the intriguing strategies deployed by *S. mansoni* to combat the host’s immune response, which may pave the way for developing new treatments.

The nematode *Onchocerca volvulus* produces GSTσ (Ov-GST1), which expresses PGD_2_ in the hypodermis, cuticle, and epicuticle upon host contact. GSTs, enzymes typically located within a cell’s cytosol, being found outside cells, as with Ov-GST1, may indicate an evolutionary adaptation. This pattern mirrors that of *F. hepatica* FhGST-S1’s GSTσ, which is released through extracellular vesicles [43]. Similar observations have been made in the cestodes *Echinococcus granulosus* (EgGST2) and *Taenia solium* (Ts24GST), where the location of GSTσ in the protoscolex and scolex’s cytosol has been confirmed. The biochemical properties and locations of these enzymes have led to suggestions of their role in drug resistance and immune-response modulation [38]. A glycosylated GSTσ, *Onchocerca ochengi* OoGST1, has been identified and characterized in the nematode, known to infect cattle and humans [71]. A glycan analysis of OoGST1 revealed the presence of α(1–3)fucose, a common carbohydrate in helminth glycoproteins, known to induce a TH2-type immune response [72]. OoGST1 can also synthesize the prostaglandins PGD_2_ and PGE_2_. Therefore, identifying GSTσ enzymes capable of producing PGD_2_ or PGE_2_ in helminths is of significant pharmacological interest because they play crucial roles in modifying the host’s immune response during parasitic infections. Thus, they are doubly strategic pharmacological targets; inhibiting them disrupts their detoxifying function and assists in negating the parasite’s immune evasion efforts.

### 3.2. GSTσ of Helminths as Vaccine Candidate

Vaccination holds numerous advantages over chemotherapy, prompting increased efforts to create vaccines against human parasites [73]. The theory is that immunizing an individual with a helminth antigen can confer protection against it and similar species. Despite varying levels of efficacy in existing research, the quest for effective vaccine antigens continues [74]. Studies have suggested that the full or specific epitopes of GSTσ can instigate an immune response, producing specific antibodies in the host, hence their consideration as vaccine candidates [75,76,77]. In particular, for *T. solium*, the immunization of mice with cytosolic GST fractions of 25 kDa and 26 kDa lessened the cysticerci burden by 90% [78]. Due to the cross-reaction that was observed with anti-Ts24GST antibodies with human GSTσ, the possibility of using specific epitopes can be explored (78).

The development of a novel purification method for *F. hepatica*’s native protein, nFhGST1, revealed increased conjugation activity with CDNB compared with its previously obtained recombinant counterpart, rFhGST1 [40,41]. This discrepancy suggests potential structural differences between the two proteins, likely stemming from post-translational modifications. Another study found that the GSTσ isolated from *F. hepatica*, nFhGST1, remained undetected by sera from goats and sheep immunized with the recombinant enzyme rFhGST-S1 [42]. However, the enzyme weakly registered after 4 weeks of infection, with a stronger detection in 12-week-old animals. This indicates an element present in the wild form of nFhGST-S that is not in the recombinant protein, likely triggering the immune-system responses of infected goats [79]. A post-translational analysis revealed that the wild protein nFhGST-S1 undergoes glycosylation and phosphorylation—processes that are absent in rFhGST-S1 [42]. Consequently, it is important to study how the production modes of proteins impact their final structure and antigenicity [80]. As of now, no suitable antigen has been found for an *F. hepatica* vaccine.

An example is Bilhvax^®^, a vaccine based on the Sh28GST antigen, which has completed both phase 1 and 2 clinical trials and is currently under phase 3 testing. Determining the crystallographic structure of this antigen has paved the way for identifying specific inhibitors [81]. An initial phase 1 clinical trial involving Sh28GST as the vaccine antigen for urinary schistosomiasis in humans revealed that it triggers a TH2-type immune response, with antibodies inhibiting Sh28GST’s enzymatic activity [82]. These findings facilitated further human research and led to phase 2 trials, which demonstrated that Bilhvax^®^, when combined with praziquantel, is safe for infected adults and children. Later, the same group reported phase 3 trial results, which showed that Bilhvax^®^ effectively controls the parasite at both the egg and adult stages, reducing the worm population by 40–70% post-infection in various animal models, including in primates. Another significant result was the vaccine’s considerable impact on the fertility of *S. haematobium*, reducing the viability of the eggs expelled by the host and, thereby, significantly blocking disease transmission. These findings led to the concept of an “anti-fertility vaccine” [76].

Another, GST-1 from *Necator americanus* (Na-GST-1) belonging to the nu class, has also been identified as a vaccine candidate, and phase 1 clinical trials have been performed in Brazil, the United States, and Gabon [83,84,85].

### 3.3. GSTσ of Helminths as Drug Target

Recently, a compound known as Ha14 was discovered, and it effectively suppresses GSTσ activity in *F. hepatica* and rFgGST-S1 in *F. gigantica* [42]. In vitro, tests demonstrated that Ha14 competently manages the adult species and diminishes egg vitality, often outperforming Triclabendazole (TCBZ), the standard reference drug. This illustrates that Ha14 is a promising candidate for controlling *Fasciola*, indicating a potential leader in the quest for analogs with superior inhibitory characteristics.

Because mammalian hosts also possess GSTs, it is important that, in the search for inhibitors of helminth GSTs, the structures of the host’s homologous GSTs are also considered in the selection of drug binding sites in which there are few common residues, as this would achieve better selectivity toward the parasite enzyme [86].

## 4. Perspectives

Vaccination is a well-acknowledged solution for controlling pathogens. Still, there are no commercial vaccines available for humans despite the identification of numerous promising helminth antigens. Helminth GSTσ antigens, which trigger a host’s humoral response, may offer a novel treatment model for these parasitic infections. Their utility lies in GSTσ’s essential role in producing PGD_2_ and PGE_2_—crucial elements at various stages of a helminth’s life cycle.

Notably, PGD_2_ plays a critical role in modulating the host’s immune system, making parasitosis easier. Additionally, GSTσ’s detoxifying property enables it to resist drugs, thus contributing to parasite survivability. As such, inhibiting this enzyme could substantially increase the parasite’s susceptibility and suppress PGD_2_ production. Developing inhibitors for GSTσ, as well as integrating them into vaccines with other cGSTs, could enhance their efficacy. Additionally, the simultaneous use of vaccines and anti-GST drugs could drastically reduce the incidence of such infectious diseases. Thus, GSTσ presents a viable target and a fresh strategy for parasite control that could be beneficial in medical applications.

## Figures and Tables

**Table 1:** Classification of the three main GST superfamilies, functions, and classes. The subfamily with the greatest diversity is the cytosolic subfamily, with 13 different classes [10,16,17,18,19,20,21,22,23].

**G S T**	**Cytosolic**	Involved in metabolization of xenobiotics, drugs, and Insecticides and in immunomodulation via the synthesis of prostaglandin D_2_	**Mammals:** Classes: alpha, mu, pi, sigma, theta, zeta, omega **Plants:** Classes: phi, tau, lambda, iota, theta, sigma, zeta **Insects and Helminths** Classes: alfa-mu, epsilon, delta, mu, sigma, omega, theta, zeta
	**Mitochondrial**	They participate in energy and lipid metabolism in the mitochondria	**Humans** Class: Kappa **Helminths** Unclassified
	**MAPEG** (Membrane-associated)	Involved in the biosynthesis of eicosanoids, glutathione, and prostaglandin E_2_	**Humans** 5-Lipoxygenase activating protein **(FLAP)** Leukotriene C4 synthase Microsomal prostaglandin E2 synthase 1 **(MPGES1)**

**Table 2:** Enzymatic activity of GSTσ from helminths, insects, and mollusks on different substrates.

Organism	GSTσ	Activity on Different Substrates (µmol/min/mg)						
		CDNB ^a^	DCNB ^b^	EAC ^c^	HEN ^d^	CHP ^e^	PC ^f^	Reference
**Helminths**								
*Ascaris galli*	n	N.D.	N.D.	260	0.5	1	Detected	[39]
*Fasciola hepatica*	rFhGST-S1	474 ± 292	N.D.	898 ± 204	645 ± 129	7080 ± 1010	Detected	[40]
	rFhGSTs1a	13,900 ± 907						[41]
	nFhGSTs1b	7360± 216	N.D.	1730 ± 212				[42]
*Onchocerca volvulus*	nOv-GST1		10	0.649± 0.015		N.D.	Detected	[43]
*Schistosoma mansoni*	rSm28GST	7270 ± 25	1580 ± 97	158 ± 97	287 ± 17	167 ± 7	Detected	[37]
*Taenia solium*	rTsMσGST	1.08	N.D.	N.D.	8.4 ± 0.4			[44]
**Insects**								
*Bombyx mori*	rbmGSTS2	0.006		0.04	5.43		Detected	[45]
*Drosophila melanogaster*	nDmGSTS1-1	0.49 ± 0.02	0.44 ± 0.01	0.24 ± 0.02	9.4 ± 0.6	0.173		[46]
*Hyphantria cunea*	nhcGST	6.99 ± 0.78			0.90 ± 0.09			[47]
*Migratory locusta*	rLmGSTs5	3890 ± 962	7.41 ± 0.96					[48]
*Phlebotomus argentipes*	rPargGSTσ	8.75			204	92.5		[49]
*Solenopsis invicta*	nSiGSTS1	87.4 ± 5.1						[50]
*Tribolium castaneum*	rTcGSTS6	3.8			176	66.7		[23]
**Mollusks**								
*Biomphalaria alexandrina*	nBaGST2	30 ± 1.5				0.60 ± 0.02		[51]
*Hyriopsis cumingii*	rHcGSTS	4.54 ± 0.08						[52]
*Loligo vulgaris*	n*L*. *vulgaris*GST	273	0.6	N.D.	17.7	1.2		[53]
*Ruditapes philippinarum*	rRpGSTσ	4.6 ± 0.17	0.28 ± 0.03	0.39 ± 0.02			N.D.	[54]

^a^ CDNB = 1,2-dichloro-4-nitrobenzene, ^b^ DCNB = 3,4-Dichloronitrobenzene, ^c^ EAC = ethacrynic acid, ^d^ HEN = 4-hydroxynonenal, ^e^ CHP = cumene hydroperoxide, ^f^ PC = prostaglandin. Origin of protein: n = native, r = recombinant.
